# *N*′-Prop­ylisonicotinohydrazide

**DOI:** 10.1107/S1600536808027955

**Published:** 2008-09-06

**Authors:** Shu-Ye Wang, Xue-Ming Song, Li-Xiang Duan

**Affiliations:** a The First Affiliated Hospital, Harbin Medical University, Harbin 150001, People’s Republic of China

## Abstract

In the title compound, C_9_H_11_N_3_O, the crystal structure is stabilized by a bifurcated inter­molecular N—H⋯(N,O) hydrogen bond and a C—H⋯O inter­action, leading to chains of mol­ecules.

## Related literature

For background on the medicinal uses of isoniazid (isonicotinic acid hydrazide, INH) and INH hydrazide–hydrazones, see: Fox & Mitchison (1975 ▶); Kucukguzel *et al.* (2003 ▶). For the synthesis, see: Deng *et al.* (2005 ▶). For bond-length data, see: Allen *et al.* (1987 ▶). 
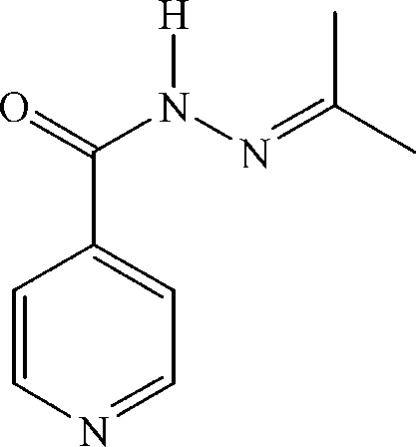


## Experimental

### 

#### Crystal data

C_9_H_11_N_3_O
*M* *_r_* = 177.21
Orthorhombic, 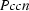

*a* = 13.010 (3) Å
*b* = 17.590 (4) Å
*c* = 8.0000 (16) Å
*V* = 1830.8 (6) Å^3^
*Z* = 8
Mo *K*α radiation
μ = 0.09 mm^−1^
*T* = 297 (2) K
0.43 × 0.28 × 0.22 mm

#### Data collection

Bruker APEXII CCD diffractometer
Absorption correction: multi-scan (*SADABS*; Bruker, 2001 ▶) *T*_min_ = 0.963, *T*_max_ = 0.981
9110 measured reflections
1634 independent reflections
986 reflections with *I* > 2σ(*I*)
*R*_int_ = 0.062

#### Refinement

*R*[*F* ^2^ > 2σ(*F* ^2^)] = 0.047
*wR*(*F* ^2^) = 0.145
*S* = 1.00
1634 reflections
125 parameters
1 restraint
H atoms treated by a mixture of independent and constrained refinement
Δρ_max_ = 0.17 e Å^−3^
Δρ_min_ = −0.14 e Å^−3^

### 

Data collection: *APEX2* (Bruker, 2004 ▶); cell refinement: *SAINT-Plus* (Bruker, 2001 ▶); data reduction: *SAINT-Plus*; program(s) used to solve structure: *SHELXS97* (Sheldrick, 2008 ▶); program(s) used to refine structure: *SHELXL97* (Sheldrick, 2008 ▶); molecular graphics: *SHELXTL* (Sheldrick, 2008 ▶); software used to prepare material for publication: *SHELXTL*.

## Supplementary Material

Crystal structure: contains datablocks I, global. DOI: 10.1107/S1600536808027955/hb2787sup1.cif

Structure factors: contains datablocks I. DOI: 10.1107/S1600536808027955/hb2787Isup2.hkl

Additional supplementary materials:  crystallographic information; 3D view; checkCIF report

## Figures and Tables

**Table 1:** Hydrogen-bond geometry (Å, °)

*D*—H⋯*A*	*D*—H	H⋯*A*	*D*⋯*A*	*D*—H⋯*A*
N2—H2*A*⋯O1^i^	0.926 (15)	2.172 (19)	3.001 (3)	149 (2)
N2—H2*A*⋯N3^i^	0.926 (15)	2.497 (16)	3.268 (2)	140.9 (19)
C9—H9*A*⋯N3^i^	0.96	2.58	3.525 (3)	167

Symmetry code: (i) 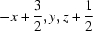.

**Fig. 1.**
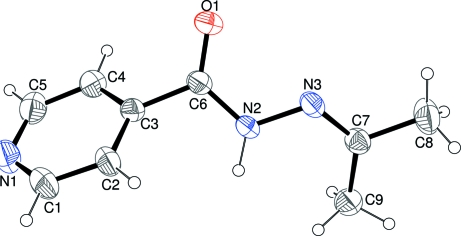
The molecular structure of (I), drawn with 30% probability displacement ellipsoids for the non-hydrogen atoms.

**Fig. 2.**
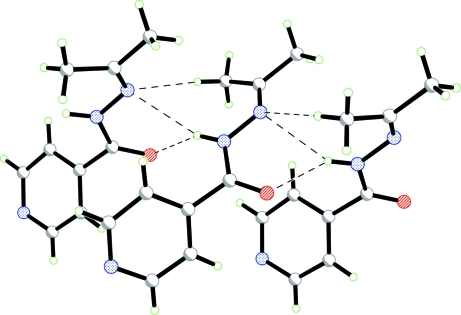
Part of a chain of molecules of (I) connected by hydrogen bonds (dashed lines).

## supplementary crystallographic information

### Comment

Isoniazid (isonicotinic acid hydrazide, INH) continues to be the most widely used chemotherapeutic agent for the treatment of tuberculosis (Fox & Mitchison, 1975). Some INH hydrazide–hydrazones were reported to have lower toxicity than hydrazides because of the blockage of the –NH_2_ group (Kucukguzel *et al.*2003). In this paper, we report the structure of the title compound, (I), (Fig. 1).

The bond lengths and angles for (I) are within their normal ranges (Allen *et al.*,  1987). The dihedral angle between the mean planes on the N1/C1–C5 ring and the O1/N2/N3/C6 grouping is 48.97 (12)°.

As shown in Fig. 2, the crystal structure is stabilized by bifurcated intermolecular N—H···(N,O) hydrogen bonds (Table 1) and C—H···O interactions leading to chains of molecules.

### Experimental

The title compound was synthesized according to the literature method (Deng *et al.*, 2005): acetone (25 mmol) and isonicotinyl hydrazine (22 mmol) were dissolved in anhydrous ethanol (40 ml) and refluxed for 5 h, and a yellow precipitate was obtained, which was recrystalized from ethanol and diethyl ether (1:1 v/v) to yield yellow blocks of (I) after two days in an ice box.

### Refinement

The N-bonded H atom was located in a difference map and freely refined. The C-bonded H atoms were placed in calculated positions with C—H = 0.93–0.96 Å and refined as riding with *U*_iso_(H) = 1.2*U*_eq_(C) or 1.5U_eq_(methyl C).

### Crystal data

**Table:**

C_9_H_11_N_3_O	*F*(000) = 752
*M**_r_* = 177.21	*D*_x_ = 1.286 Mg m^−^^3^
Orthorhombic, *P**c**c**n*	Mo *K*α radiation, λ = 0.71073 Å
Hall symbol: -P 2ab 2ac	Cell parameters from 1634 reflections
*a* = 13.010 (3) Å	θ = 2.0–25.1°
*b* = 17.590 (4) Å	µ = 0.09 mm^−^^1^
*c* = 8.0000 (16) Å	*T* = 297 K
*V* = 1830.8 (6) Å^3^	Block, yellow
*Z* = 8	0.43 × 0.28 × 0.22 mm

### Data collection

**Table:**

Bruker APEXII CCD diffractometer	1634 independent reflections
Radiation source: fine-focus sealed tube	986 reflections with *I* > 2σ(*I*)
graphite	*R*_int_ = 0.062
φ and ω scans	θ_max_ = 25.1°, θ_min_ = 2.0°
Absorption correction: multi-scan (SADABS; Bruker, 2001)	*h* = −15→15
*T*_min_ = 0.963, *T*_max_ = 0.981	*k* = −19→20
9110 measured reflections	*l* = −6→9

### Refinement

**Table:**

Refinement on *F*^2^	Primary atom site location: structure-invariant direct methods
Least-squares matrix: full	Secondary atom site location: difference Fourier map
*R*[*F*^2^ > 2σ(*F*^2^)] = 0.047	Hydrogen site location: difmap (N-H) and geom (C-H)
*wR*(*F*^2^) = 0.145	H atoms treated by a mixture of independent and constrained refinement
*S* = 1.00	*w* = 1/[σ^2^(*F*_o_^2^) + (0.070*P*)^2^ + 0.2547*P*] where *P* = (*F*_o_^2^ + 2*F*_c_^2^)/3
1634 reflections	(Δ/σ)_max_ < 0.001
125 parameters	Δρ_max_ = 0.18 e Å^−^^3^
1 restraint	Δρ_min_ = −0.14 e Å^−^^3^

### Special details

**Table:**

Geometry. All e.s.d.'s (except the e.s.d. in the dihedral angle between two l.s. planes) are estimated using the full covariance matrix. The cell e.s.d.'s are taken into account individually in the estimation of e.s.d.'s in distances, angles and torsion angles; correlations between e.s.d.'s in cell parameters are only used when they are defined by crystal symmetry. An approximate (isotropic) treatment of cell e.s.d.'s is used for estimating e.s.d.'s involving l.s. planes.
Refinement. Refinement of *F*^2^ against ALL reflections. The weighted *R*-factor *wR* and goodness of fit *S* are based on *F*^2^, conventional *R*-factors *R* are based on *F*, with *F* set to zero for negative *F*^2^. The threshold expression of *F*^2^ > σ(*F*^2^) is used only for calculating *R*-factors(gt) *etc*. and is not relevant to the choice of reflections for refinement. *R*-factors based on *F*^2^ are statistically about twice as large as those based on *F*, and *R*- factors based on ALL data will be even larger.

### Fractional atomic coordinates and isotropic or equivalent isotropic displacement parameters (Å^2^)

**Table:**

	*x*	*y*	*z*	*U*_iso_*/*U*_eq_	
C1	0.5785 (2)	0.3888 (2)	0.6946 (4)	0.0774 (9)	
H1	0.5509	0.4037	0.7967	0.093*	
C2	0.6065 (2)	0.44485 (16)	0.5836 (3)	0.0615 (7)	
H2	0.5975	0.4959	0.6102	0.074*	
C3	0.64780 (16)	0.42398 (14)	0.4339 (3)	0.0475 (6)	
C4	0.65648 (19)	0.34815 (15)	0.4012 (3)	0.0599 (7)	
H4	0.6825	0.3317	0.2992	0.072*	
C5	0.6266 (2)	0.29651 (16)	0.5193 (4)	0.0699 (8)	
H5	0.6338	0.2451	0.4945	0.084*	
C6	0.67852 (18)	0.48038 (13)	0.3061 (3)	0.0493 (6)	
C7	0.84373 (18)	0.63336 (14)	0.2827 (3)	0.0494 (6)	
C8	0.8682 (2)	0.69467 (16)	0.1616 (4)	0.0771 (9)	
H8A	0.8188	0.6941	0.0725	0.116*	
H8B	0.9358	0.6866	0.1168	0.116*	
H8C	0.8659	0.7430	0.2174	0.116*	
C9	0.91433 (19)	0.62320 (16)	0.4257 (3)	0.0620 (8)	
H9A	0.8751	0.6165	0.5263	0.093*	
H9B	0.9573	0.6673	0.4365	0.093*	
H9C	0.9565	0.5792	0.4073	0.093*	
N1	0.58839 (18)	0.31505 (15)	0.6654 (3)	0.0767 (8)	
N2	0.73950 (15)	0.53574 (12)	0.3610 (2)	0.0517 (6)	
N3	0.76506 (16)	0.59328 (11)	0.2493 (2)	0.0544 (6)	
O1	0.64977 (13)	0.47481 (10)	0.1615 (2)	0.0676 (6)	
H2A	0.7650 (17)	0.5349 (14)	0.4690 (16)	0.072 (8)*	

### Atomic displacement parameters (Å^2^)

**Table:**

	*U*^11^	*U*^22^	*U*^33^	*U*^12^	*U*^13^	*U*^23^
C1	0.092 (2)	0.087 (2)	0.0534 (18)	−0.0139 (18)	0.0222 (15)	0.0038 (16)
C2	0.0764 (18)	0.0595 (17)	0.0486 (16)	−0.0105 (14)	0.0130 (13)	0.0062 (13)
C3	0.0441 (14)	0.0593 (17)	0.0391 (13)	−0.0091 (11)	−0.0025 (11)	0.0023 (11)
C4	0.0596 (17)	0.0651 (19)	0.0552 (17)	−0.0069 (14)	0.0043 (12)	−0.0020 (14)
C5	0.0658 (18)	0.0616 (18)	0.082 (2)	−0.0076 (14)	−0.0046 (16)	0.0095 (16)
C6	0.0495 (14)	0.0609 (16)	0.0375 (14)	−0.0071 (12)	−0.0004 (11)	0.0039 (12)
C7	0.0465 (14)	0.0565 (16)	0.0453 (14)	−0.0012 (12)	0.0043 (11)	0.0032 (11)
C8	0.0697 (18)	0.078 (2)	0.084 (2)	−0.0169 (16)	0.0030 (16)	0.0294 (16)
C9	0.0562 (15)	0.0726 (18)	0.0574 (17)	−0.0112 (13)	−0.0062 (13)	0.0025 (13)
N1	0.0825 (17)	0.078 (2)	0.0697 (18)	−0.0147 (14)	0.0044 (13)	0.0211 (14)
N2	0.0602 (13)	0.0628 (14)	0.0321 (11)	−0.0156 (11)	−0.0034 (9)	0.0091 (10)
N3	0.0579 (13)	0.0647 (14)	0.0406 (12)	−0.0108 (11)	−0.0030 (9)	0.0147 (10)
O1	0.0759 (13)	0.0883 (14)	0.0387 (10)	−0.0241 (10)	−0.0097 (8)	0.0064 (9)

### Geometric parameters (Å, °)

**Table:**

C1—N1	1.324 (4)	C6—N2	1.331 (3)
C1—C2	1.376 (4)	C7—N3	1.271 (3)
C1—H1	0.9300	C7—C8	1.484 (3)
C2—C3	1.363 (3)	C7—C9	1.478 (3)
C2—H2	0.9300	C8—H8A	0.9600
C3—C4	1.364 (3)	C8—H8B	0.9600
C3—C6	1.480 (3)	C8—H8C	0.9600
C4—C5	1.367 (4)	C9—H9A	0.9600
C4—H4	0.9300	C9—H9B	0.9600
C5—N1	1.312 (4)	C9—H9C	0.9600
C5—H5	0.9300	N2—N3	1.391 (2)
C6—O1	1.220 (3)	N2—H2A	0.926 (10)
			
N1—C1—C2	124.2 (3)	N3—C7—C9	126.6 (2)
N1—C1—H1	117.9	C8—C7—C9	117.4 (2)
C2—C1—H1	117.9	C7—C8—H8A	109.5
C3—C2—C1	118.6 (3)	C7—C8—H8B	109.5
C3—C2—H2	120.7	H8A—C8—H8B	109.5
C1—C2—H2	120.7	C7—C8—H8C	109.5
C4—C3—C2	117.7 (2)	H8A—C8—H8C	109.5
C4—C3—C6	120.0 (2)	H8B—C8—H8C	109.5
C2—C3—C6	122.2 (2)	C7—C9—H9A	109.5
C5—C4—C3	119.6 (3)	C7—C9—H9B	109.5
C5—C4—H4	120.2	H9A—C9—H9B	109.5
C3—C4—H4	120.2	C7—C9—H9C	109.5
N1—C5—C4	124.0 (3)	H9A—C9—H9C	109.5
N1—C5—H5	118.0	H9B—C9—H9C	109.5
C4—C5—H5	118.0	C5—N1—C1	116.0 (2)
O1—C6—N2	123.7 (2)	C6—N2—N3	117.57 (19)
O1—C6—C3	121.2 (2)	C6—N2—H2A	120.6 (15)
N2—C6—C3	115.1 (2)	N3—N2—H2A	121.8 (15)
N3—C7—C8	116.0 (2)	C7—N3—N2	117.42 (19)

### Hydrogen-bond geometry (Å, °)

**Table:**

*D*—H···*A*	*D*—H	H···*A*	*D*···*A*	*D*—H···*A*
N2—H2A···O1^i^	0.93 (2)	2.17 (2)	3.001 (3)	149 (2)
N2—H2A···N3^i^	0.93 (2)	2.50 (2)	3.268 (2)	141.(2)
C9—H9A···N3^i^	0.96	2.58	3.525 (3)	167

Symmetry codes: (i) −*x*+3/2, *y*, *z*+1/2.
